# Nanostructured Manganite Films Grown by Pulsed Injection MOCVD: Tuning Low- and High-Field Magnetoresistive Properties for Sensors Applications

**DOI:** 10.3390/s22020605

**Published:** 2022-01-13

**Authors:** Voitech Stankevic, Nerija Zurauskiene, Skirmantas Kersulis, Valentina Plausinaitiene, Rasuole Lukose, Jonas Klimantavicius, Sonata Tolvaišienė, Martynas Skapas, Algirdas Selskis, Saulius Balevicius

**Affiliations:** 1 Center for Physical Sciences and Technology, 02300 Vilnius, Lithuania; nerija.zurauskiene@ftmc.lt (N.Z.); skirmantas.kersulis@ftmc.lt (S.K.); valentina.plausinaitiene@chf.vu.lt (V.P.); jonas.klimantavicius@ftmc.lt (J.K.); martynas.skapas@ftmc.lt (M.S.); algirdas.selskis@ftmc.lt (A.S.); saulius.balevicius@ftmc.lt (S.B.); 2 Faculty of Electronics, Vilnius Gediminas Technical University, 10223 Vilnius, Lithuania; sonata.tolvaisiene@vilniustech.lt; 3 Faculty of Chemistry and Geosciences, Vilnius University, 03225 Vilnius, Lithuania; 4 IHP–Leibniz-Institut für Innovative Mikroelektronik, 15236 Frankfurt (Oder), Germany; lukose@ihp-microelectronics.com

**Keywords:** MOCVD technology, nanostructured thin films, colossal magnetoresistance, low field magnetoresistance, manganite films, magnetic field sensors

## Abstract

The results of colossal magnetoresistance (CMR) properties of La_0.83_Sr_0.17_Mn_1.21_O_3_ (LSMO) films grown by pulsed injection MOCVD technique onto various substrates are presented. The films with thicknesses of 360 nm and 60 nm grown on AT-cut single crystal quartz, polycrystalline Al_2_O_3_, and amorphous Si/SiO_2_ substrates were nanostructured with column-shaped crystallites spread perpendicular to the film plane. It was found that morphology, microstructure, and magnetoresistive properties of the films strongly depend on the substrate used. The low-field MR at low temperatures (25 K) showed twice higher values (−31% at 0.7 T) for LSMO/quartz in comparison to films grown on the other substrates (−15%). This value is high in comparison to results published in literature for manganite films prepared without additional insulating oxides. The high-field MR measured up to 20 T at 80 K was also the highest for LSMO/quartz films (−56%) and demonstrated the highest sensitivity *S* = 0.28 V/T at *B* = 0.25 T (voltage supply 2.5 V), which is promising for magnetic sensor applications. It was demonstrated that Mn excess Mn/(La + Sr) = 1.21 increases the metal-insulator transition temperature of the films up to 285 K, allowing the increase in the operation temperature of magnetic sensors up to 363 K. These results allow us to fabricate CMR sensors with predetermined parameters in a wide range of magnetic fields and temperatures.

## 1. Introduction

During recent decades, the increasing demand in magnetic sensing has resulted in extensive investigations on materials exhibiting magnetic properties, which allow us to decrease sensor dimensions and to increase sensitivity and ranges of operating temperature and magnetic field. The commercially available magnetic sensors based on magnetoresistive xMR effects (anisotropic AMR, tunneling TMR, and giant GMR) revealed increased sensitivity in comparison with widely used Hall sensors [1,2]. As a result, the magnetic field sensors market analysis and forecast report [3] predicted an increase in the magnetoresistive sensors market at the expense of Hall sensors. The colossal magnetoresistive effect (CMR) is also promising for future sensors development technologies [4]. It has to be mentioned that each type of area of application has specific requirements for the device specifications, sensor size, magnetic field, temperature ranges of operation, and sensor’s accuracy. Therefore, the development of various sensors technologies that could be fast adopted from laboratory-scale to commercial production are of great importance.

Recently, it has been demonstrated that nanostructured La_1-x_Sr_x_MnO_3_ (La-Sr-Mn-O) manganite films exhibiting a CMR effect can be used for the development of magnetic sensors, which are capable to measure the magnitude of pulsed magnetic fields in very small volumes. Such sensors were used for the measurement of the magnetic diffusion processes in railguns [5] and the magnetic field distribution in non-destructive pulsed-field magnets [6]. Manganite films reveal paramagnetic–ferromagnetic phase transition at a Curie temperature and exhibit negative colossal MR phenomenon. Over the last few decades, many research groups have become interested in so-called extrinsic magnetoresistance [7] related to electron transport phenomena in grain boundaries of polycrystalline manganites, since these promise a large MR values in much wider temperature range in comparison with epitaxial films exhibiting significant MR only in the vicinity of phase transition temperature. However, the MR decreases with an increase in temperature in a paramagnetic state and saturates at high fields. It was shown that sensors based on nanostructured La-Sr-Mn-O manganite films can measure magnetic field magnitude independently on field direction (so-called B-scalar sensors) [8]. The complexity of manganite oxides allows them to tune their electric and magnetic properties over a wide range of temperatures and magnetic fields. It can be performed by variation of chemical composition, film thickness and deposition temperature, and disorder induced by different nanostructure of polycrystalline films.

One of the main features of polycrystalline (nanostructured) manganites is the so-called low field magnetoresistance (LFMR) effect. It is associated with the existence of a large number of grain boundaries (GBs) having disordered structure and reduced magnetic properties [9]. LFMR was explained by spin polarized tunneling of charge carriers through GBs [10,11]. It is more pronounced at low temperatures and weak fields and determines the sensitivity of nanostructures and thin films to magnetic field. The major focus has been made on fabrication of various types of grain boundaries providing an energy barrier for spin-polarized tunneling of charge carriers. Specially grown thin film junctions (La-Sr-Mn-O/SrTiO_3_/La-Sr-Mn-O) [12], composite films of manganite with insulating oxides such as ZnO or CeO [13,14], synthesis of manganite nanoparticles [15], and other methods were proposed to increase the LFMR, and as a result, magnetic sensing possibilities. It has to be noted that, for wide range of applications, sensors measuring magnetic fields up to several Tesla are desirable. Therefore, the combination of LFMR and high-field effects are of great interest. It was demonstrated that the pulsed injection (PI) metalorganic chemical vapor deposition (MOCVD) technique allows easy tuning of magnetoresistive properties of thin nanostructured La-Sr-Mn-O manganite films over the wide range of temperatures and magnetic fields by changing deposition temperature and the film thickness [16]. The glass-ceramics substrate used in the mentioned work [16] allowed to grow macroscopically homogeneous nanostructured films with column-shaped grains, with the dimensions determining the magnetoresistance and its anisotropy properties of these films. For further understanding of a role of various grain boundaries and their influence on film properties, the investigations of the films grown on different substrates have to be performed. Numerous studies have been performed on characterization of lanthanum manganite films grown on various substrates by using a magnetron DC sputtering and laser ablation methods [17,18]. However, these physical deposition techniques are usually used as laboratory-scale methods, which are difficult to apply for industrial applications.

In this paper, we present a comprehensive study of investigation of magnetoresistive properties of nanostructured lanthanum manganite films grown by PI MOCVD technique on different substrates and demonstrate the possibility to use these films for the development of magnetic field sensors with predetermined parameters. 

## 2. Experimental Details

### 2.1. Film Preparation

The nanostructured manganite La_1-x_Sr_x_Mn_y_O_3_ (LSMO) thin films were grown on different substrates by PI MOCVD technique. Different types of substrates were used during the same deposition process: AT-cut single-crystal quartz (monocrystalline SiO_2_), Si/SiO_2_-1000 nm with 1000 nm-thick amorphous SiO_2_ layer, and polycrystalline Al_2_O_3_. All substrates were purchased from MTI Corporation (Richmond, CA USA). AT-cut quartz and Si/SiO_2_ substrates were chosen due to their wide range of applications in electronics, and while polycrystalline Al_2_O_3_ was selected due to possibility to increase operation temperature range of LSMO/Al_2_O_3_ sensors [19]. Moreover, different crystalline structure of the substrates could result in different film growth mechanisms, which could influence the magnetoresistive properties of the LSMO films. Two different thicknesses (360 nm and 60 nm) of the films were deposited at a constant deposition temperature of 750 °C. The La(thd)3, Sr(thd)2, and Mn(thd)3 (thd is 2,2,6,6-tetramethyl-3,5-heptandionate) synthesized in the laboratory and dissolved in toluene were used as precursors. The microdoses (~3 mg) of an organic solution containing a dissolved mixture of organometallic precursors were injected with a 2 Hz frequency. The chemical composition of the solution was used the same for all films (La_0.8_Sr_0.2_Mn_0.73_) and was optimized to have Sr content in the thicker films x~0.17; however, for thinner films, the Sr content was obtained lower than expected. It has to be mentioned that the films were grown with Mn excess *y* = Mn/(La + Sr) = 1.21. After flash evaporation at ~270 °C of the microdoses, the vapor mixture was transported by an Ar + O_2_ (5:1) gas flow (~95 l/h) towards the heated substrate in the reaction chamber. The total pressure of Ar + O_2_ gases was 5 Torr. The thickness of the films was controlled by changing deposition time and was estimated by a profilometer after deposition process. The determined growth rate was about 28 nm/min. After the growth, the films were annealed for 10 min. at the same temperature (750 °C) in a pure O_2_ atmosphere followed by slow cooling to 350 °C temperature with ~4.7 °C/min rate. In the following sections, the substrate influence on the properties of the La_0.83_Sr_0.17_Mn_1.21_O_3_ films will be demonstrated. 

### 2.2. Characterization

Scanning Electron Microscope (SEM) (Hitachi SU70) was used to study the surface morphology of the films. The composition of the films was determined by inductively coupled plasma high-resolution mass spectrometry (ICP-MS). The crystal structure of the samples was investigated using a transmission electron microscope (TEM) (Tecnai G2 F20 X-TWIN) with an energy-dispersive X-ray spectrometer (EDAX).

For the electric transport and magnetoresistance measurements, the Ag electrodes with a Cr sublayer were thermally deposited and post-annealed at 450 °C for 1 h in an Ar atmosphere. A twisted pair cable was used to minimize measurement error in pulsed magnetic fields due to induced electromotive force. The resistivity and low-field magnetoresistance were measured in the temperature range of *T* = (5–310) K and (25–300) K, respectively, using a closed-cycle helium gas cryocooler (Janis 4K) and electromagnet (up to 0.8 T). The high-field magnetoresistance measurements were performed in pulsed magnetic fields up to 22 T in the temperature range of (80–363) K using a pulsed magnetic field generator based on capacitor bank discharge through a non-destructive coil. For the statistical analysis, several samples cut from the same film were measured.

## 3. Results and Discussion

### 3.1. Morphology and Microstructure of LSMO Films

**Figure 1:**
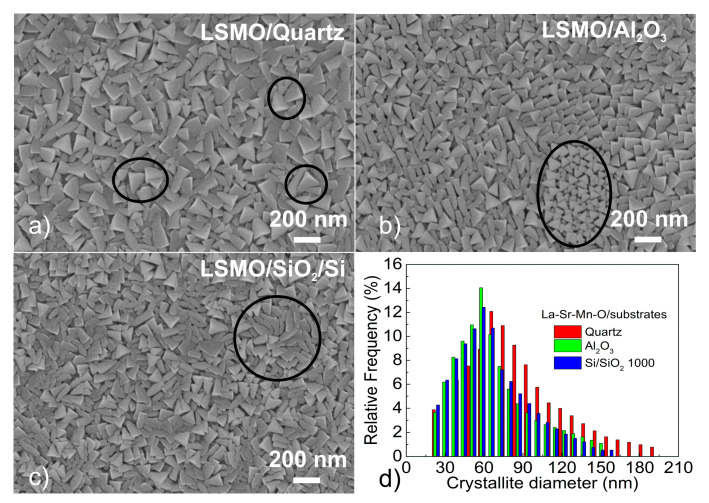
SEM surface images of 360 nm-thick LSMO films grown on different substrates: AT-cut single-crystal quartz (**a**), polycrystalline Al_2_O_3_ (**b**), and amorphous Si/SiO_2_ substrates (**c**). Distribution of crystallite dimensions (D) for films on different substrates (**d**).

SEM images of surface morphology of 360 nm-thick LSMO films grown on different substrates are presented in Figure 1. The surface of the films contains triangular shape crystallites with some polygonal-shaped grains. The surface morphology of the films grown on monocrystalline quartz (Figure 1a) is different in comparison to the films grown on polycrystalline Al_2_O_3_ (Figure 1b) and amorphous Si/SiO_2_ substrates (Figure 1c). Figure 1d presents a histogram of the crystallite size distribution obtained from SEM images. One can see that crystallites grown on quartz have larger dimensions (most frequent crystallite maximum at 70 nm) in comparison with other films (maximum at 60 nm) and is more spread indicating a larger amount of different crystallite dimensions. It is probably caused by the crystalline surface of quartz and large crystallites of Al_2_O_3_ substrates. For these substrates, in the initial stage of films growth, nuclei are more oriented and larger than on amorphous SiO_2_. Moreover, it has to be noted that polycrystalline Al_2_O_3_ substrates are made from an ingot, which consists of grains of about 2 μm in dimensions. When cutting into disks, a surface of substrates is formed by grains of different sizes and crystallographic planes. Therefore, during the initial growth process, the LSMO film starts to grow on different planes and size of Al_2_O_3_ crystallites; thus, SEM images show some islands in which shape of the LSMO crystallites is different from the other parts of the films (see Figure 1b). SEM images of thinner films (60 nm) showed much smaller crystallites.

**Figure 2:**
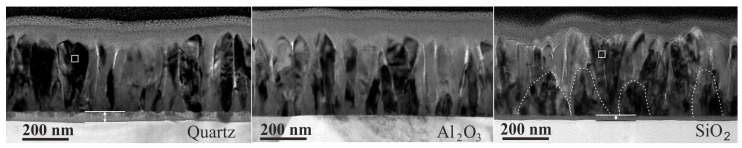
TEM image of the film grown on quartz, Al_2_O_3_ and amorphous Si/SiO_2_ substrates.

To understand the growth peculiarities, TEM images of grown films were analyzed. The low-magnification cross-sectional TEM images of the thicker films (360 nm) are shown in Figure 2. As can be seen, all films consist of columns, which are spread throughout the whole film thickness with their long axis arranged perpendicular to the substrate. The typical column width in all films is about 50–70 nm on the upper side and 20–50 nm near the substrate.

**Figure 3:**
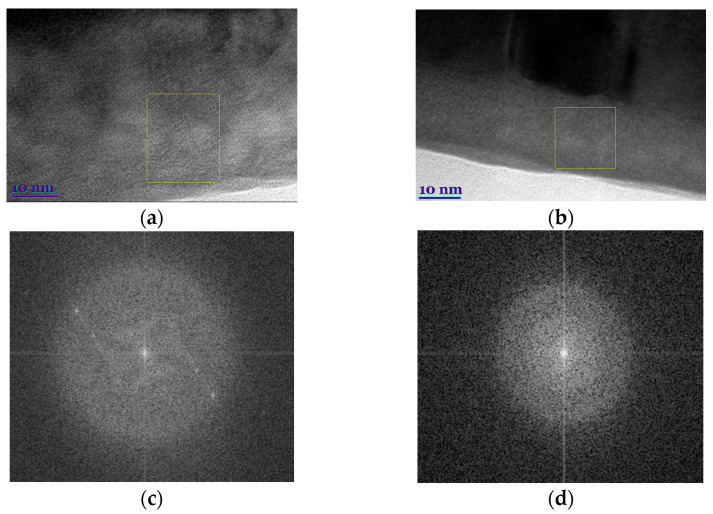
HR TEM image of an interface between the LSMO film and substrate: quartz (**a**) and Si/SiO_2_ (**b**). Fast Fourier transformation (FFT) patterns of the marked square area (**c**,**d**), respectively.

The main difference between the films grown on amorphous Si/SiO_2_ and single-crystal quartz or polycrystalline Al_2_O_3_ (composed of 2–3 µm crystallites) is that on Si/SiO_2_ together with long columns spread throughout the whole film thickness large clusters consisting of smaller crystallites co-exist (see Figure 2). Furthermore, the contrast of the TEM images shows that films grown on quartz and Si/SiO_2_ substrates have an intermediate gray layer between the substrate and the films. The thickness of this layer is about 40 nm for the films grown on quartz substrate and about 20 nm for films on Si/SiO_2_ substrate. The high-resolution TEM images (Figure 3a) and fast Fourier transformation (FFT) patterns (Figure 3c) show that the intermediate layer of film grown on quartz has a crystalline structure with an amorphous medium. However, the lattice parameters deduced from the FFT of the HREM image do not match with those associated with LSMO structure. In the contrast, the intermediate layer of the film grown on Si/SiO_2_ substrates consists only of the amorphous phase (see Figure 3b,d).

**Figure 4:**
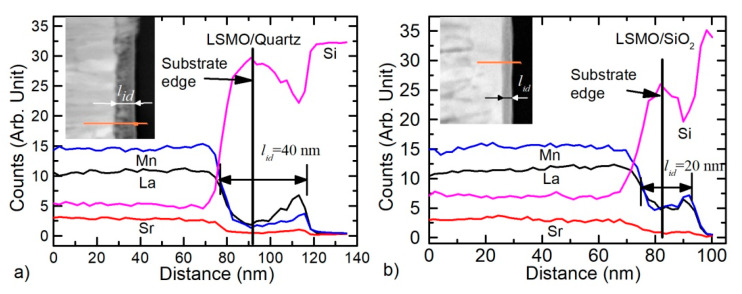
Normalized TEM-EDS line profiles of the elements of 360 nm-thick LSMO films grown on quartz (**a**) and Si/SiO_2_ (**b**) substrates. Insets—cross-sectional STEM Z-contrast image with marked scanning lines.

The role of the substrates on the film interface was examined by analyzing the distribution of the main elements across the film thickness. The TEM-EDS line profiles of these elements and cross-sectional STEM Z-contrast images of the films grown on quartz and Si/SiO_2_ substrates are shown in Figure 4. The initial position of the substrate surface is shown in these figures by the black line.

From the depth profiles, one can see that the mutual diffusion takes place in both cases and intermediate layer of the films is the glass-like phase that contains such elements as Si, Sr, Mn, La, and O. Furthermore, if the silicon diffusion into films has a profile similar to Gaussian distribution, then the distribution profile of the film atoms in substrates is more complex. The concentration of atoms diffused from the film has a certain minimum near the substrate edge and maximum at the junction (border) between the substrate-intermediate layer (see Figure 4). Moreover, the concentration of Mn atoms in this intermediate layer is less than the concentration of La ones (opposite relation than in the film). Perhaps it is caused by the different diffusion rates of these atoms into the substrate. However, the issue of the presence of Si atoms in the depth of the films remains open; other methods are needed to determine it. However, In-Bo Shim et al. [20] and Young-Min Kang et al. [21] showed that during annealing of LSMO film deposited on Si/SiO_2_ substrate, the Si was detected all across the LSMO layer. In these works, the compositional analysis was performed by Auger Electron Spectroscopy.

**Figure 5:**
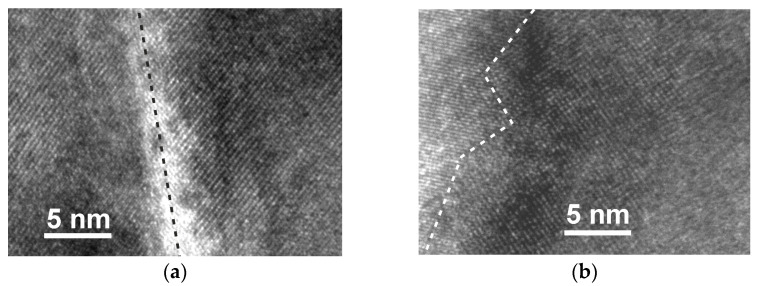
HR TEM image of an interface between the two adjacent LSMO grains of films grown on quartz (**a**) and Si/SiO_2_ (**b**) substrates.

The HR TEM image of the crystalline part of the film grown on different substrates was studied to evaluate the quality of crystallites and grain boundaries. Figure 5 presents an interface of two adjacent LSMO grains of the film grown on quartz and Si/SiO_2_ substrates (positions of these places are shown in Figure 2). In both cases, crystalline phases of LSMO grains with grain boundary regions can be observed. However, for the films grown on quartz, the border between the grains is more perfect than for films grown on Si/SiO_2_. For example, in Figure 5a, the two neighboring crystallites are tilted only by a few degrees, and the boundary between them is straight with the width of the grain boundary of about 1 nm. In opposite, the boundary between crystallites grown on Si/SiO_2_ substrate have a zigzag shape with a width of about 2–3 nm.

**Figure 6:**
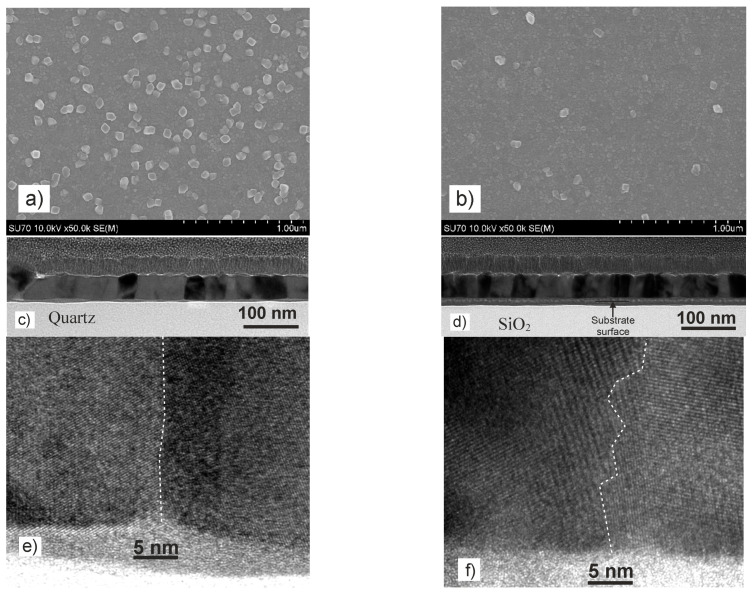
SEM surface images of 60 nm-thick LSMO films grown on different substrates: quartz (**a**), Si/SiO_2_ substrates (**b**). Low-resolution TEM images of films grown on quartz (**c**) and Si/SiO_2_ (**d**). High-resolution TEM images of an interface between intermixed layer (near the substrate) and two adjacent LSMO grains ((**e**)—film on quartz, (**f**)—film on Si/SiO_2_). The dotted lines show the grain boundaries.

Additionally, thin LSMO films with thickness of 60 nm were studied. Figure 6 presents the surface morphology and interface studies. One can see (Figure 6a,b) that the surface of these films mainly consists of small polygonal-shape grains with a characteristic size of about 30 nm, while some “large” (~70 nm) polygonal-shape grains were also observed.

The low-resolution TEM images show that the film on quartz (see Figure 6c) consists of monocrystalline grains with a clear faceted surface. The “large” grains that are visible in the SEM images are crystals with an orientation other than the rest of the film with the crystallite column thickness of about 80 nm (see in the left corner of Figure 6c). Films grown on Si/SiO_2_ substrate (see Figure 6d) also consist of separate crystallites; however, they are not as perfect as on the quartz substrate. Moreover, the films grown on quartz and amorphous Si/SiO_2_ substrates have intermediate layer as was observed for the thick LSMO films, which was confirmed by high-resolution TEM.

The high-resolution TEM images in Figure 6e,f also show an interface between the intermediate layer and LSMO films. One can see that two adjacent LSMO grains of the films grown on the quartz substrate (see Figure 6e) have a perfect structure with a strict boundary between crystallites. The width of the grain boundary in this case is less than 1 nm. At the same time, crystallites on a Si/SiO_2_ substrate have a less perfect structure, with a zigzag interface between them and a wider boundary. This is probably due to the different crystal nature of the substrate. In the first case, due to the monocrystalline structure of the quartz substrate, nucleation of oriented crystallites takes place. Then, during their growth and coalescence, well-oriented crystallites are formed. In the second case, crystallite growth occurs on an amorphous substrate. Therefore, the nuclei of initial crystallite are randomly oriented, and after their coalescence, smaller crystallites with a random orientation are formed. 

**Figure 7:**
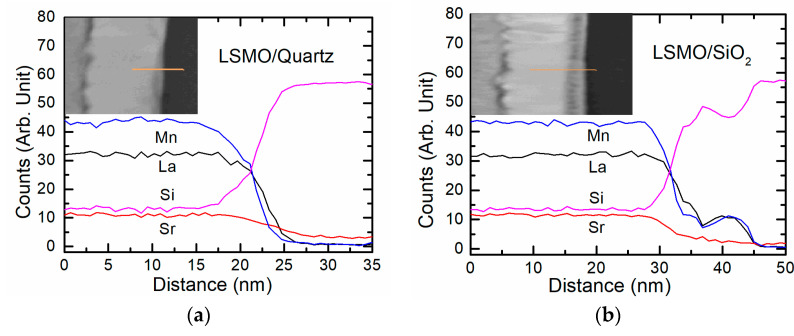
Normalized TEM-EDS line profiles of the elements of 60 nm thick LSMO grown on quartz (**a**) and Si/SiO_2_ (**b**) substrates. Insets—cross-sectional STEM Z-contrast image with marked scanning lines.

Moreover, at this growth stage of LSMO films, the intermediate layer of 15 nm was more pronounced on Si/SiO_2_ substrate, whereas on quartz, this layer was only 5 nm thick. The TEM-EDS diffusion profiles of the main elements (see Figure 7) confirmed the mutual diffusion. The same diffusion lines of the elements (La, Sr, Mn) towards substrate and the opposite direction (Si towards the film) were determined for the thinner LSMO films in comparison to the thicker films grown on Si/SiO_2_ substrates (see Figure 4b and Figure 7b). However, in contrast to the thick films, the diffusion profile of La, Mn, and Sr into the quartz substrate is different; it has the same Gaussian-like shape as the Si diffusion into the LSMO film (see Figure 7a).

It can be concluded that, at the initial stage of the growth, due to the high temperature of the substrate (750 °C), the diffusion of film atoms into the amorphous Si/SiO_2_ substrate and Si atoms into the film takes place much more intensively than into the quartz substrate. This is probably due to the amorphous nature of Si/SiO_2_ substrate. We assume that during the further growth of the film, the diffusion of Si into the film is blocked by a high concentration of Mn and La atoms that diffused into the opposite direction—from the film into the substrate (see the maximum of Mn and La in Figure 4). This conclusion can be drawn by comparing the thickness of the intermediate layer in thin and thick films, since with an increase in the film thickness to 360 nm, the total interdiffusion layer increased only to 20 nm. In the case of film growth on single-crystal quartz substrates, diffusion of film atoms into the substrate, and vice versa, occurs more slowly, and deeper penetration of film atoms into the quartz substrate occurs during further film growth. Additionally, one should pay attention to the interface between the intermediate layer and the films. It can be seen that this interface is not flat, and the gray intermediate layer penetrates deeper into the film in the grain boundary region (see Figure 6e). This testifies to an easier diffusion of Si atoms to the film through these boundaries. However, due to the limited resolving power of TEM-EDS, it is difficult to conclude something unequivocally about the penetration of Si atoms into the films through whole film thickness, and more sensitive methods are needed. Other methods such as XPS depth profiles should be used. Furthermore, contrary to the Si-based substrates, no noticeable mutual diffusion of elements was found in the films grown on Al_2_O_3_ substrate. Perhaps this is due to the different binding energies of the atoms in these substrates.

### 3.2. Resistivity of Nanostructured LSMO Films: Dependence on Film Thickness and Ambient Temperature

**Figure 8:**
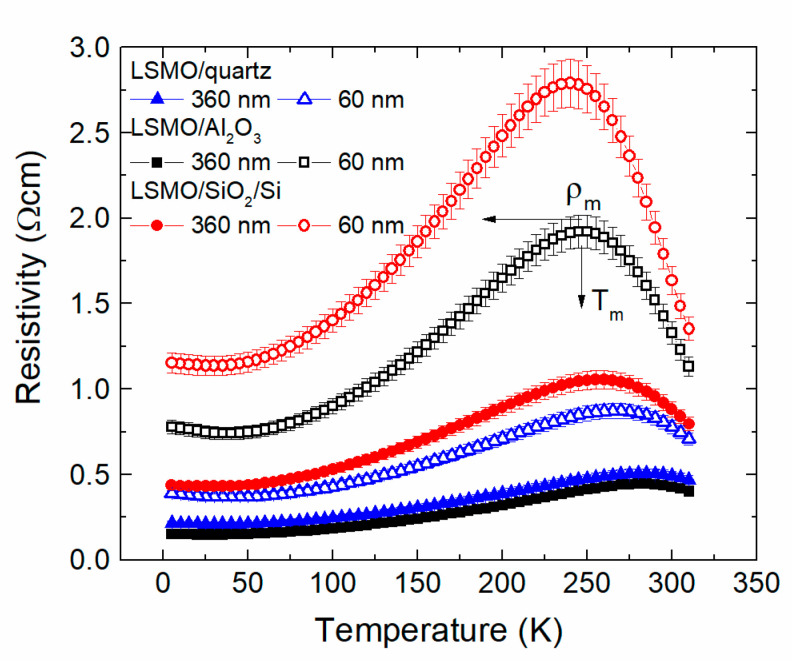
Resistivity vs. temperature dependences of LSMO films with different thicknesses (60 nm and 360 nm) grown on different substrates: quartz, Al_2_O_3_, and Si/SiO_2_. The error bar shows scatter of the results of several samples fabricated from the same film.

Figure 8 presents resistivity *ρ* of thin (60 nm) and thicker (360 nm) LSMO films grown on different substrates. The measurement error was less than 1%; thus, the error bar shows the scatter of the results of several samples fabricated from the same film. One can see that films exhibit a transition from metal-like to an insulator-like resistivity dependence on temperature at a certain critical temperature *T*_m_ corresponding to resistivity maximum *ρ*_m_. However, the electrical transport properties of the films grown on different substrates and having different thicknesses are significantly different. The *T*_m_ and *ρ*_m_ values are summarized in Table 1. One can see that the thinner the film, the lower the metal-insulator transition temperature *T*_m_ and higher the maximal resistivity *ρ*_m_. It is related to microstructure of the films. The polycrystalline film could be imagined as a net of crystallites having perfect structure and disordered grain boundaries between them (see Figure 2 and Figure 6c,d). The conducting mechanism in crystallites, as in monocrystalline-doped manganites, is controlled by double exchange mechanism between manganese ions: Mn^3+^–O^2−^–Mn^4+^. However, the resistivity of polycrystalline films is mostly determined by the grain boundary material and its relative quantity. Therefore, depending on the disorder of grain boundaries and relative quantity of their material, the resistivity of the films can differ in a wide range. The thinner films grow in smaller crystallites (see Figure 6a–d) and have more disordered grain boundary material in the same volume as compared with thicker films. The thicker films have larger crystallites and less grain boundary material, and as a result, the resistivity is decreased in comparison to the thinner films. 

The influence of the substrate on the *T*_m_ and *ρ*_m_ values is also determined by initial growth of the films as can be seen from TEM images (see Figure 2 and Figure 6c,d). Despite the diffusive interlayer for films grown on single-crystal quartz, the crystallite columns grow thicker and more homogeneous in comparison with the films on the other substrates which leads to the lowest resistivity (*ρ*_m_ = 0.5 Ωcm) and the highest insulator–metal transition temperature (*T*_m_ =285 K). The highest *ρ*_m_ and lowest *T*_m_ was observed for films grown on amorphous Si/SiO_2_.

It has to be noted that such high *T*_m_ values (285 K) for nanostructured LSMO films were obtained mainly due to Mn excess in the films: Mn/(La + Sr) = 1.21. Such Mn excess causes increased *T*_m_ values, especially for LSMO deposited on quartz and Al_2_O_3_ substrates, which suggests the possibility of increasing the operation temperature of magnetic sensors based on these films. Recently, it was demonstrated that the chemical composition of LSMO films with Mn excess has a great influence on their resistivity maximum temperature and magnetoresistive properties [19]. The mentioned study [19] of similar films grown on Al_2_O_3_ substrates revealed the following *T*_m_ values: 240 K, 250 K, and 270 K for LSMO films with Mn excess of 1.05, 1.10, and 1.15, respectively. It is worth mentioning that an increase in Mn of >1 causes non-stoichiometry of the films, and it is difficult to control the homogeneity of the films having only one LSMO phase. For this reason, to ensure single LSMO phase of the films, we tried to keep Mn excess no higher than ~1.2. As mentioned before, the chemical composition of the films studied in this work was La_0.83_Sr_0.17_Mn_1.21_O_3_.

### 3.3. Magnetoresistance of Nanostructured LSMO Films

#### 3.3.1. Low-Field Magnetoresistance

**Figure 9:**
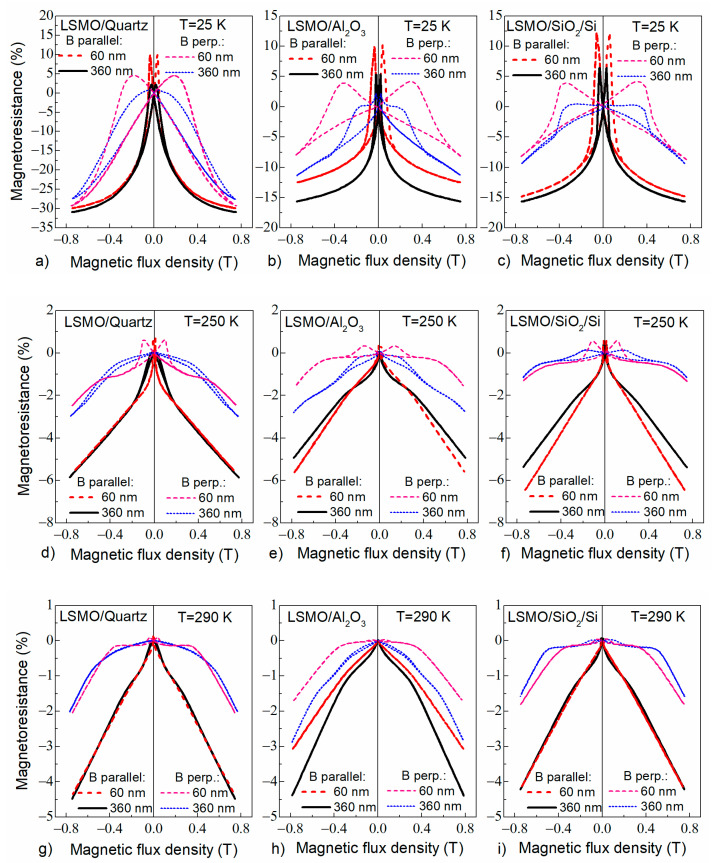
Low-field magnetoresistance dependences on magnetic flux density for different thickness films (60 nm and 360 nm) grown on different substrates and measured at various ambient temperatures: 25 K, 250 K, 290 K.

The low-field magnetoresistance dependences on magnetic flux density (*B*) measured up to 0.75 T at different temperatures are presented in Figure 9 for two field configurations: *B* parallel (*B*_||_) or perpendicular (*B*_⊥_) to the film plane. The measurement temperatures corresponding to the ferromagnetic state (25 K), close to metal–insulator transition (250 K) and paramagnetic state (290 K) were chosen. The *MR* was defined as follows: *MR* = 100% × [*ρ* (*B*)−*ρ* (0)]/*ρ* (0), where *ρ* (*B*) and *ρ* (0) are field and zero field resistivity, respectively. The main features of the *MR* are more pronounced at low temperatures. Figure 9a–c show *MR*(*B*) dependences at 25 K. For *B*_||_, one can see sharp positive *MR* changes at a low magnetic field, maxima of which are attributed to the coercive field of the films. Additionally, a sharp increase in the negative magnetoresistance (the large decrease in electrical resistance) at low fields and a slower background negative *MR* in high field is observed with increase in the magnetic field. These effects are usually called low-field magnetoresistance (*LFMR*) and high-field magnetoresistance (*HFMR*), respectively. 

In our investigated samples, the *LFMR* was strongly dependent on the substrate used for film growth. One can see that, for LSMO/quartz, the *LFMR* is about 31% at 0.7 T for 360 nm film and 305 for 60 nm thick films, while for LSMO/SiO_2_/Si films, it is 15% and 145, and for LSMO/Al_2_O_3_ films, 15% and 125, respectively.

It should be noted that the *LFMR* is usually not observed in epitaxial films or monocrystalline manganites [10] and is attributed to the films with a large number of grain boundaries having noncollinear spin structure in which transport (tunneling) across the grain boundaries dominates. *LFMR* is usually more pronounced at low temperatures and depends on the thickness and type of grain boundaries, because they decouple the neighboring ferromagnetic (FM) grains and provide an energy barrier for spin-polarized tunneling of electrons. However, it was shown by Hwang et al. [10] that an *LFMR* of ≥30% was never achieved with natural grain boundaries in polycrystalline manganite samples. For example, Lee et al. [11] showed that magnetoconductance (*MC*) could achieve maximal value of only 33.3% (corresponds to the 25% of *MR*) for polycrystalline La_0.67_Sr_0.33_MnO_3_ films. Obviously, the *LFMR* (0.7 T) values for LSMO/quartz films are much higher in comparison to the proposed theoretical limit by Lee et al. [11]. This result is promising for application of LSMO films for magnetic sensing purposes. 

To increase the *LFMR* values, vertically aligned nanocomposite manganites films with artificially introduced oxides are used [14]. Moreover, Sadhu et al. [15] compared the results on *LFMR* found in published literature for various manganite oxide films and their composites with various oxides. The comparison shows that the largest *LFMR* values could be obtained by using nanostructures LSMO/SrTiO_3_/LSMO or composites (for example, LSMO/ZnO). Gao et al. [14] obtained large increase in *LFMR* for LCMO:CeO_2_ thin films by introducing insolating CeO_2_ phase in the La-Ca-Mn-O films, which generates the magnetic disordered phase boundary and serves as the energy barrier, enhancing the spin-fluctuation suppression effect and resulting in enhanced *LFMR* in the LCMO. The *LFMR* values obtained for LSMO/quartz films in the present study appeared to be the largest in comparison with manganite films without the introduction of additional oxide phases [15]. This could be explained as follows. The single-crystal quartz substrate determines the largest crystallite dimensions of the grown films in comparison to other substrates, which ensures high quality crystallites connected through thin straight grain boundaries (see Figure 5a), which allows spin-polarized tunneling without scattering as it occurs at thicker and more disordered GBs of the films grown on the other substrates. In addition, the self-formation of the interdiffusion layer (see Section 3.1) between the substrate and the film, acting as an insulating layer, can have an influence on the increased *LFMR* values at low temperatures.

At a higher temperature, a decrease in *LFMR* values is caused by a decrease in the contribution of the pure tunneling effect through GBs and increased spin scattering in these disordered regions. 

For *B*_⊥_, one can see a large (up to 0.4 T) demagnetization field caused by the “shape” effect (film thickness is much lower than dimension of the film in plane). However, one can notice that for the films with the same thickness, the demagnetization field differs depending on the substrate used. We assume that the demagnetization factor depends not only on the geometrical dimensions of the film but also on the crystallite dimensions, crystallites and grain boundary structure, and on their magnetic properties. When the film is composed of larger crystallites (as in the thicker films), the magnetic interaction of the crystallites in film plane is good, and the demagnetization field is higher (~0.4 T). For thin films, the crystallite dimensions are much smaller, and the relative amount of grain boundary material is larger; therefore, the long-range magnetic interaction of the crystallites along the film plane is reduced. Thus, the demagnetization field is smaller. 

For B-scalar magnetic sensors application, the magnetoresistance anisotropy (*MRA*) is very important. The *MRA* was defined as relative change of the *MR* in respect to the field orientation: *MRA* = 100% × (*MR*_||_−*MR*_⊥_)/*MR*_||_. Due to the demagnetization effect, the *MRA* of the films is significant at low fields and decreases with the increase in the field. Low-field *MRA* values of the 360 nm-thick films grown on different substrates are shown in Table 2. The presented results were obtained at different measured temperatures by applying a magnetic field of 0.7 T. One can see that the film on the polycrystalline Al_2_O_3_ has lowest *MRA* (40%) at room temperature.

**Figure 10:**
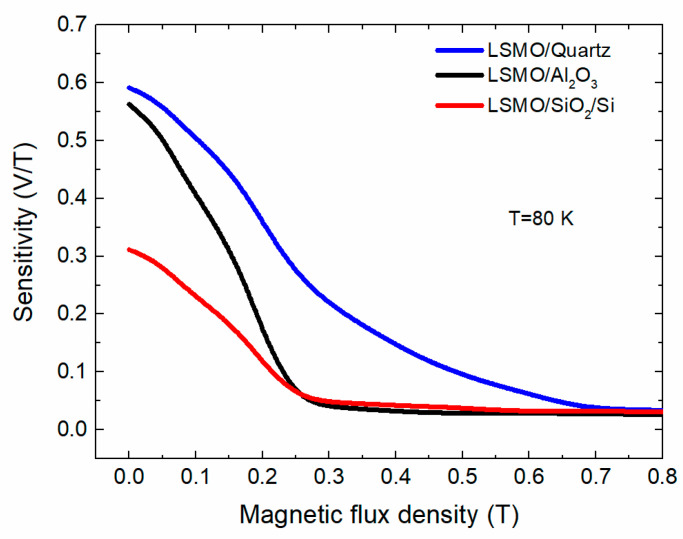
Sensitivity dependences on magnetic flux density (*B*_||_) for LSMO sensors produced from films grown on different substrates. Measured using voltage supply of *V*_s_ = 2.5 V.

Other important parameter for magnetic sensor development is the sensitivity to magnetic field. In our experiments, the sensor with a resistance of approximately 6 kΩ was connected in series with ballast resistor of the same nominal value, and a voltage of 2.5 V was supplied using a voltage source. The sensitivity *S* = (δ*V*/δ*B*) is defined as the absolute value of the voltage change across the sensor’s (sample’s) resistance *R* with the change of the magnetic field by 1 T. Figure 10 presents the sensitivity dependences on magnetic flux density at *T* = 80 K for LSMO sensors produced from films grown on various substrates.

It can be seen from the graph that the highest sensitivity at low field exhibits the film grown on quartz substrate. The sensitivity of LSMO/quartz sensors was 0.6 V/Tat *B* = 0.02 T; it decreased to 0.28 V/T at *B* = 0.25 T and became almost constant (~0.04 V/T) at *B* > 0.7 T. The sensors prepared from films grown on Al_2_O_3_ and Si/SiO_2_ substrates had lower sensitivity. Such increase in the sensitivity of LSMO/quartz sensors is a result of higher low-field magnetoresistance effect of these films. The abrupt change in sensitivity at low fields is also a result of sharp *LFMR* effect (see Figure 9). It has to be noted that, starting from 2.5 T, the *S* was almost constant (~18 mV/T) in the whole measured range up to 20 T (see section below for high-field measurements).

#### 3.3.2. High-Field Magnetoresistance

**Figure 11:**
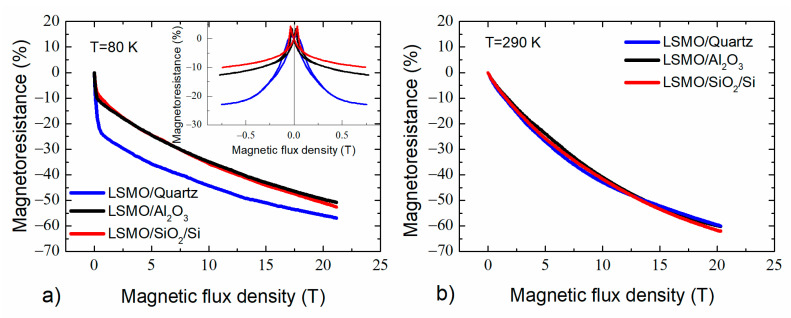
Magnetoresistance dependences on magnetic flux density at (**a**) *T*= 80 K and (**b**) *T* = 290 K for 360 nm thick films grown on quartz, Al_2_O_3_ and Si/SiO_2_ substrates. The inset—*MR* vs. *B* measured by using a permanent field electromagnet.

Magnetoresistance dependences on magnetic flux density for 360 nm thick films grown on quartz, Al_2_O_3_, and Si/SiO_2_ substrates are shown in Figure 11. The measurements were performed using pulsed fields up to ~20 T at ambient temperatures (a) *T*= 80 K and (b) *T* = 290 K. For comparison, the inset in Figure 11a presents *LFMR* measured by different technique (in a permanent field using an electromagnet).

At low temperatures (80 K), the films grown on quartz exhibit highest magnetoresistance magnitude (26% and 56%), both at low (*B* = 0.7 T) and high (*B* = 20 T) magnetic fields, respectively. This is mostly related to the highest *LFMR*, as discussed in the previous section. At room temperature, the *MR* magnitude was similar for all films and high enough at 20 T (~60%). However, for the evaluation of high-field magnetoresistive properties of the LSMO films, the crystallites of high structural quality, and disordered material of grain boundaries should be taken into consideration. Usually, for monocrystalline or epitaxial films, the *MR* saturates at moderate fields (<10 T) [22] when all magnetic moments of manganese are aligned with the field direction. For polycrystalline films (or nanostructured as in our case), very high fields are required to align disordered regions of GBs that are in paramagnetic state at room temperature. Therefore, the *MR* is not saturated up to 20 T. As can be seen in Figure 11, the higher tendency for *MR* saturation shows the film grown on single-crystal quartz substrate. However, this tendency is not significant, because, due to thin interdiffusion layer between the quartz substrate and the film (see Section 3.1), the nanostructured film was grown instead of epitaxial one. The sensitivity at room temperature of the samples (sensors) prepared from these films was 40 mV/T and 18 mV/T at 0.7 T and 20 T, respectively (*V*_s_ = 2.5 V).

**Figure 12:**
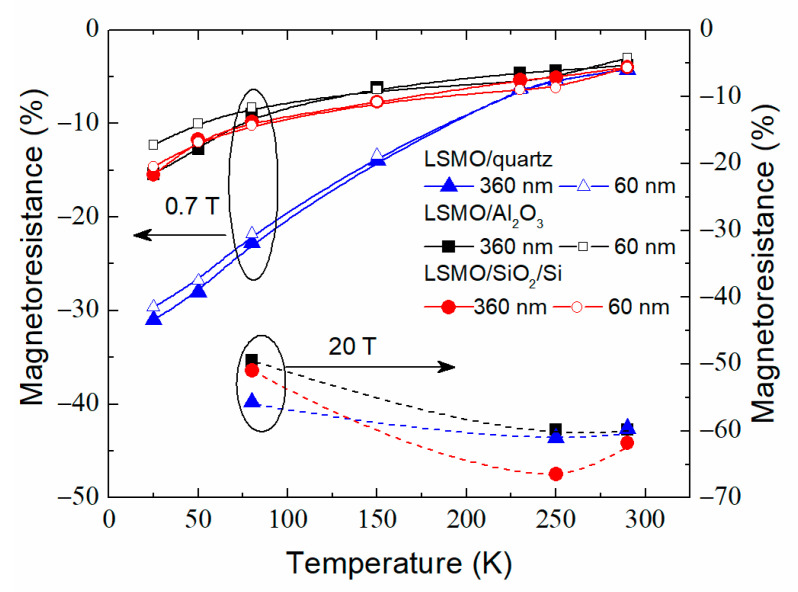
Magnetoresistance dependences on temperature at 0.7 T (left scale) and 20 T (right scale) for LSMO films grown on different substrates.

Figure 12 summarizes the temperature dependences of the *MR* measured at *B* = 0.7 T (left scale) and 20 T (right scale) for LSMO films grown on different substrates. One can see that, at low temperatures, the LSMO/quartz films are preferable for sensors applications. At room temperature (290 K), the *MR* is similar for all films independently on thickness and used substrate. The highest *MR* magnitude obtained at 250 K for LSMO grown on Si/SiO_2_ could be explained by the fact that the *MR* usually is highest in the vicinity of the metal–insulator transition temperature. In this case, *T*_m_ of this film was 260 K, which is close to the measurement temperature (250 K).

**Figure 13:**
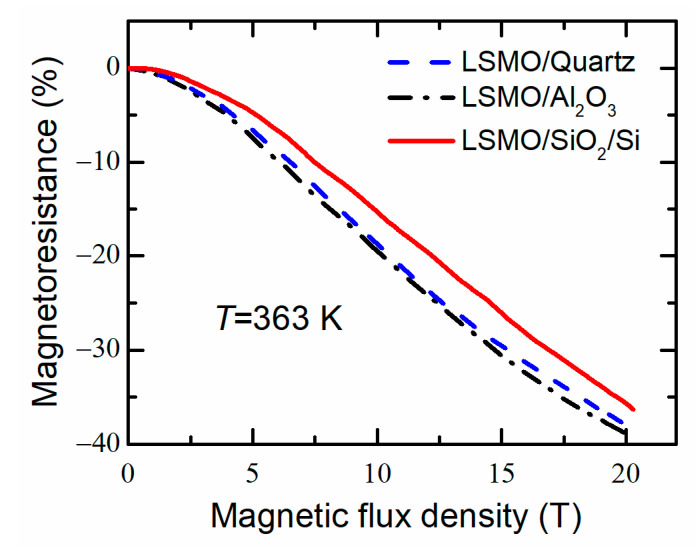
Magnetoresistance dependences on magnetic flux density at *T* = 363 K for 360 nm thick films grown on quartz, Al_2_O_3_ and Si/SiO_2_ substrates.

Figure 13 shows the *MR* measured at higher than room temperature (363 K) for LSMO films grown on different substrates. One can see that, at higher temperatures, the *MR* at low fields is reduced; however, it still remains significant at high fields (*MR* magnitude (~35–39%) at 20 T). The largest *MR* values are obtained for the films grown on quartz and Al_2_O_3_ substrates. The LSMO/quartz and LSMO/Al_2_O_3_ films show highest and almost the same magnetoresistance values at the whole magnetic flux density range up 20 T. Smaller *MR* values for film grown on Si/SiO_2_ substrate could be explained by a reduced metal–insulator transition temperature (*T*_m_ = 260 K) in comparison with films on other substrates (285 K). Thus, at 363 K, the LSMO on Si/SiO_2_ substrate is in paramagnetic state and higher fields are required to align magnetic moments of Mn.

Therefore, the LSMO films with Mn excess of Mn/(La + Sr) = 0.21 grown on quartz and Al_2_O_3_ substrates could be used for the development of magnetic field sensors operating up to 90 °C (363 K). The evaluated sensitivity of such sensors was ~18 mV/T in the range of (5–20) T, when *V*_s_ = 2.5 V.

**Figure 14:**
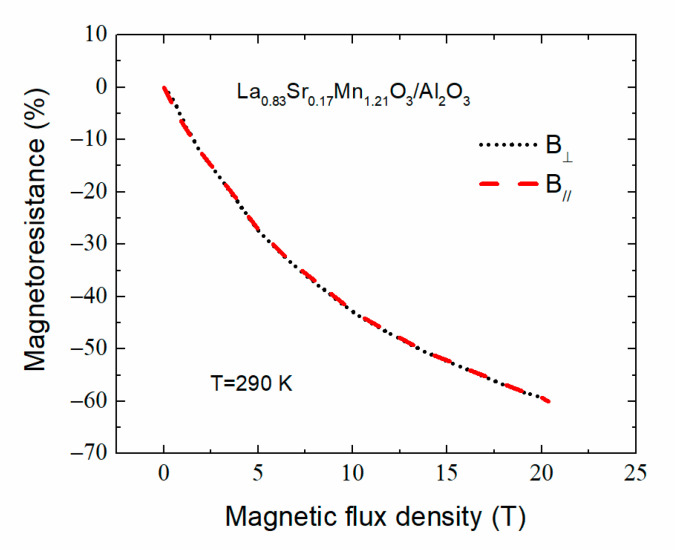
Magnetoresistance of La_0_._83_Sr_0_._17_Mn_1_._21_O_3_ film deposited on Al_2_O_3_ substrate vs. magnetic induction in high magnetic field at 290 K temperature.

As mentioned in Section 3.3.1, another important parameter for B-scalar magnetic sensors application is magnetoresistance anisotropy. Figure 14 presents the magnetoresistance of 360 nm-thick La_0_._83_Sr_0_._17_Mn_1_._21_O_3_ film deposited on Al_2_O_3_ substrate measured at 290 K temperature up to 20 Tesla. There is only a slight mismatch between the *MR* in *B*_||_ and *B*_⊥_ configuration at *B* < 1 T; above this value, the curves practically overlap. This confirms that, in high magnetic fields, the *MRA* of investigated nanostructured LSMO films is negligible.

## 4. Conclusions

The choice of the substrate for deposition of the nanostructured La_1-x_Sr_x_Mn_y_O_3_ manganite films has a great influence on the main parameters (resistivity, magnetoresistance and its anisotropy) of these films in ferromagnetic phase at low magnetic fields (<0.7 Tesla). Films with thickness of 360 nm grown on single-crystal quartz exhibit two times higher low-field magnetoresistance (−31% at *T* = 25 K, when *B* = 0.7 T) in comparison with the films grown on polycrystalline Al_2_O_3_ and amorphous Si/SiO_2_-1000 substrates (−15%). This difference could be explained by: (i) a more orderly structure of LSMO films grown on quartz with column-shaped crystallites spread through the whole film thickness and separated by thin grain boundary regions or (ii) the interdiffusive layer formation at the quartz and manganite boundary/interface.

The high-field (20 Tesla) magnetoresistance values of nanostructured LSMO films grown on different substrates are similar. This could be explained as follows: the high field influences mainly magnetic ordering of Mn ions in disordered grain boundary regions, while magnetic ordering (magnetoresistance) of different quality crystallites obtained during deposition on different substrates has tendency of saturation in the fields of around 10 Tesla. Increases in Mn content above stoichiometric level increase the temperature of the metal–insulator transition and increase the magnetoresistance of the films, especially in high magnetic fields. The highest *MR* values (−38% at *B* = 20 T and *T* = 363 K) were found for the LSMO films with Mn excess of 1.21 deposited on the polycrystalline Al_2_O_3_ substrate. These LSMO/Al_2_O_3_ films had lowest magnetoresistance anisotropy (40%) at room temperature at low-field (B = 0.7 T) and negligible *MRA* (less than 1%) in high fields.

It was concluded that nanostructured lanthanum manganite films grown on AT-cut single-crystal quartz or polycrystalline Al_2_O_3_ substrates could be used for the development of magnetic field sensors with predetermined parameters for operation at low or high temperatures, respectively.

## Figures and Tables

**Table 1:** *T*_m_ and *ρ*_m_ values for thick (360 nm) and thin (60 nm) LSMO films grown on different substrates: quartz, Al_2_O_3_ and Si/SiO_2_.

Substrate	Film Thickness, nm	*ρ*_m_, Ω cm	*T*_m_, K
Quartz	360	0.5	285
	60	0.9	265
Al_2_O_3_	360	0.44	285
	60	1.9	250
Si/SiO_2_	360	1.1	260
	60	2.8	240

**Table 2:** Magnetoresistance anisotropy (*MRA*) of the 360 nm-thick films grown on different substrates, measured at ambient temperatures *T* = 25 K, 250 K, and 290 K at magnetic flux density of 0.7 T.

Substrate	*T* = 25 K	*T* = 250 K	*T* = 290 K
Quartz	14%	50%	61%
Al_2_O_3_	30%	46%	40%
Si/SiO_2_	43%	80%	71%
